# Supplementary material for: Microbial dynamics and pulmonary immune responses in COVID-19 secondary bacterial pneumonia
Source: Nat Commun. 2024 Oct 29;15:9339. doi: 10.1038/s41467-024-53566-x (PMC11522429; doi:10.1038/s41467-024-53566-x)
Supplement: Supplementary file 1 — Supplementary Information [file 41467_2024_53566_MOESM1_ESM.pdf]

# Supplementary Materials

## Supplementary Tables:

**Supplementary Table 1**. Clinical and demographic features of cohort.

**Supplementary Table 2.** Clinical and demographic features of cohort analyzed by metatranscriptomics.

**Supplementary Table 3.** Relationship between *mecA* detection and phenotypic susceptibility to methicillin in *S. aureus* cultured isolates from patients with 2°BP.

**Supplementary Table 4.** Relationship between *CTX-M* detection and phenotypic susceptibility to ceftriaxone in cultured *Enterobacteriaceae* isolates from patients with 2°BP.

**Supplementary Table 5.** Concordance between AMR gene detection in tracheal aspirate and nasal swabs.

**Supplementary Table 6.** Associations between AMR gene detection and in-hospital patient mortality.

## Supplementary Figures:

**Supplementary Figure 1**. Changes in lower respiratory microbiome alpha diversity over time.
**Supplementary Figure 2.** Beta diversity comparing tracheal aspirate samples from 2°BP versus No-BP patients, by Jaccard index.
**Supplementary Figure 3.** Alpha diversity based on days of steroid receipt, days of mechanical ventilation, SARS-CoV-2 viral load and bacterial mass.
**Supplementary Figure 4.** Beta diversity comparing tracheal aspirate samples from 2°BP versus No-BP patients, adjusted for days of steroid receipt, days of mechanical ventilation, SARS-CoV-2 viral load and bacterial mass.
**Supplementary Figure 5**. Differential bacterial abundance based on 2°BP status, limited to patients who received steroids.
**Supplementary Figure 6**. SARS-CoV-2 viral load based on 2°BP status.
**Supplementary Figure 7.** Functional analysis of bacterial metabolic pathways based on 2°BP status.
**Supplementary Figure 8.** Pathogen rank plots of all tracheal aspirate samples.
**Supplementary Figure 9.** Pathogen mass plots of all tracheal aspirate samples.
**Supplementary Figure 10.** Pathogen normalized mass plots of all tracheal aspirate samples.
**Supplementary Figure 11**. Comparative persistence of 2°BP pathogens in tracheal aspirate samples.
**Supplementary Figure 12.** Pathogen rank plots of nasal swab and tracheal aspirate samples.
**Supplementary Figure 13.** Pathogen mass plots of nasal swab and tracheal aspirate samples.
**Supplementary Figure 14.** Pathogen normalized mass plots of nasal swab and tracheal aspirate samples.

**Supplementary Figure 15.** Beta diversity comparing nasal swab to tracheal aspirate samples in A) 2°BP patients and B) No-BP patients.

**Supplementary Figure 16**. Per-patient identification of pathogen-associated antimicrobial resistance genes.

**Supplementary Figure 17.** Antimicrobial resistance (AMR) genes detected in patients with *Pseudomonas aeruginosa* 2°BP.

**Supplementary Table 1. Clinical and demographic features of cohort.** Continuous variables (age, symptom onset to admission, WHO ordinal score) were analyzed using two-sided Wilcoxon tests. Categorical variables (sex assigned at birth, race, comorbidities, treatment) was analyzed using Fisher's exact tests (>25% of cells contained <5 patients). [+]Statistical analyses exclude American Indian/Alaska Native, Native Hawaiian/Pacific Islander groups due to absence from >1 cells. [×]0-2 patients per group had missing data from comorbidities reported; the total N of patients with complete data is shown for each category. *N = 42 2°BP and N = 40 No-BP patients with available symptom data. [#]Limited to baricitinib and tocilizumab. P value of two-sided Wilcoxon test comparing mortality rates between 2°BP vs No-BP patients is 3.0e-5.

| | 2°BP | No-BP | P-value (2°BP vs No-BP) |
|---|---|---|---|
| **N** | 44 | 41 | |
| **Age in years, median (IQR)** | 62.9 (50.5, 72.5) | 54.7 (44.2, 62.9) | 0.12 |
| **Female (%)** | 16 (36.4%) | 15 (36.6%) | 1.00 |
| **Race (%)[+]** | | | 0.72 |
| American Indian/Alaska Native | 1 (2.3%) | 0 (0.0%) | - |
| Black | 5 (11.4%) | 5 (12.2%) | - |
| Asian | 5 (11.4%) | 2 (4.9%) | - |
| Native Hawaiian/Pacific Islander | 0 (0.0%) | 1 (2.4%) | - |
| White | 10 (22.7%) | 8 (19.5%) | - |
| Other/Multiple | 23 (52.3%) | 25 (61.0%) | - |
| **Hispanic ethnicity (%)** | 25 (56.8%) | 23 (56.1%) | 1.00 |
| **Comorbidities (%)[×]** | | | |
| Autoimmune | 3/43 (7.0%) | 7/41 (17.1%) | 0.19 |
| Cancer | 3/42 (7.1%) | 4/41 (9.8%) | 0.71 |
| COPD/Asthma | 8/42 (19.0%) | 13/40 (32.5%) | 0.21 |
| DM | 20/43 (46.5%) | 22/40 (55.0%) | 0.75 |
| HTN | 21/41 (47.7%) | 19/40 (47.5%) | 0.86 |
| Obesity | 27/44 (61.4%) | 29/41 (70.7%) | 0.49 |
| Solid organ transplant | 3/44 (6.8%) | 5/41 (12.2%) | 0.47 |
| **Baseline IS meds (%)** | 5 (11.4%) | 8 (19.5%) | 0.37 |
| **Received any COVID-19 vaccines prior to admission** | 4 (9.1%) | 5 (12.2%) | 0.61 |
| **Admission date to 2°BP (median, IQR)** | 7.5 (3-15.3) | - | - |
| **Ventilator to 2°BP (median, IQR)** | 6.5 (3-11.5) | - | - |
| **Symptom onset to hospital admission (median, IQR)*** | 7.0 (4.0-12.8) | 7.0 (3.0-10.0) | 0.46 |
| **WHO ordinal score at arrival** | 7 (5-7) | 7 (5-7) | 0.59 |
| **Treated with any steroids during hospitalization (%)** | 43 (97.7%) | 34 (82.9%) | 0.026 |
| **Treated with any steroids prior to 2°BP diagnosis (%)** | 41 (93.2%) | - | - |
| **Days of steroids prior to 2°BP diagnosis (median, IQR)** | 7 (3.8-9.0) | - | - |
| **Treated with non-steroid immunosuppressants (%)[#]** | 6 (13.6%) | 7 (17.1%) | 0.76 |
| **Days of antibiotic treatment during first week of hospitalization (median, IQR)** | 3.0 (1.8-7.0) | 4.0 (2.0-5.0) | 0.83 |
| **Treated with any antibiotics during hospitalization** | 44 (100.0%) | 41 (100.0%) | - |
| **Mortality (%)** | 21 (47.7%) | 3 (7.3%) | <0.0001 |

**Supplementary Table 2. Clinical and demographic features of cohort analyzed by metatranscriptomics.** Continuous variables (age, symptom onset to admission, WHO ordinal score) were analyzed using two-sided Wilcoxon tests. Categorical variables (sex assigned at birth, race, comorbidities, treatment) was analyzed using Fisher's exact tests (>25% of cells contained <5 patients). *N = 27 2°BP and N = 29 No-BP patients with available symptom data, [+]Statistical analyses exclude American Indian/Alaska Native, Native Hawaiian/Pacific Islander groups due to absence from >1 cells. [×]0-2 patients per group had missing data from comorbidities reported; the total N of patients with complete data is shown for each category. *N = 26 2°BP and N = 28 No-BP patients with available symptom data. [#]Limited to baricitinib and tocilizumab. P value of two-sided Wilcoxon test comparing mortality rates between 2°BP vs No-BP patients is 9.5e-5.

| | **2°BP** | **No-BP** | **P-value (2°BP vs No-BP)** |
|---|---|---|---|
| **N** | 27 | 29 | |
| **Age in years, median (IQR)** | 63.3 (52.2-74.7) | 51.1 (47.2-62.6) | 0.032 |
| **Female (%)** | 9 (33.3%) | 9 (31.0%) | 1.0 |
| **Race (%)[+]** | | | 0.91 |
| American Indian/Alaska Native | 1 (3.7%) | 0 (0.0%) | - |
| Black | 2 (7.4%) | 4 (13.8%) | - |
| Asian | 1 (3.7%) | 1 (3.4%) | - |
| Native Hawaiian/Pacific Islander | 0 (0.0%) | 1 (3.4%) | - |
| White | 5 (18.5%) | 4 (13.8%) | - |
| Other/Multiple | 18 (66.7%) | 19 (65.5%) | - |
| **Hispanic ethnicity (%)** | 19 (70.4%) | 17 (58.6%) | 0.41 |
| **Comorbidities (%)[×]** | | | |
| Autoimmune | 2/26 (7.7%) | 4/29 (13.8%) | 0.67 |
| Cancer | 2/25 (8.0%) | 2/29 (6.9%) | 1.0 |
| COPD/Asthma | 6/26 (23.1%) | 9/29 (31.0%) | 0.56 |
| DM | 13/26 (50.0%) | 15/28 (53.6%) | 1.0 |
| HTN | 12/25 (48.0%) | 13/29 (44.8%) | 1.0 |
| Obesity | 8/27 (29.6%) | 8/29 (27.6%) | 1.0 |
| Solid organ transplant | 1/27 (3.7%) | 3/29 (10.3%) | 0.61 |
| **Baseline IS meds (%)** | 2 (7.4%) | 5 (17.2%) | 0.42 |
| **Received any COVID-19 vaccines prior to admission** | 3 (11.1%) | 3 (10.3%) | 0.99 |
| **Admission date to 2°BP (median, IQR)** | 5.0 (3-11.0) | - | - |
| **Ventilator to 2°BP (median, IQR)** | 6.0 (3.0-10.5) | - | - |
| **Symptom onset to hospital admission (median, IQR)*** | 7.0 (4.0-11.8) | 7.0 (4.8-10.3) | 0.68 |
| **WHO ordinal score at arrival** | 7 (5.5-7.0) | 7 (5.0-7.0) | 0.85 |
| **Treated with any steroids during hospitalization (%)** | 27 (100.0%) | 23 (79.3%) | 0.011 |
| **Treated with any steroids prior to timepoint samples (%)** | 25 (92.6%) | 19 (65.5%) | 0.021 |
| **Days of steroids prior to timepoint samples (median, IQR)** | 7.0 (3.5-9.0) | 2.0 (0.0-6.0) | 0.0027 |
| **Treated with non-steroid immunosuppressants[#] (%)** | 2 (7.4%) | 5 (17.2%) | 0.42 |
| **Days of antibiotics prior to timepoint samples (median, IQR)** | 3 (1.5 -5.0) | 2.0 (1.0-7.0) | 0.85 |
| **Treated with any antibiotics during hospitalization** | 27 (100.0%) | 29 (100.0%) | 1.0 |
| **Mortality (%)** | 15 (55.6%) | 2 (6.9%) | <0.0001 |

**Supplementary Table 3. Relationship between *mecA* gene detection and susceptibility to methicillin in patients with cultured *S. aureus* isolates (N = 10).** Detection of *mecA* in tracheal aspirate or nasal swab samples within -7/+7 days of 2°BP are compared with phenotypic methicillin/nafcillin/oxacillin susceptibility of *S. aureus* isolates cultured from tracheal aspirate. Two patients had *S. aureus* isolates for which phenotypic susceptibility was not performed (No data).

| | | **Clinical Susceptibility** | | | **Calculated test performance** | | | |
|---|---|---|---|---|---|---|---|---|
| | | **Methicillin-S** (N = 6) | **Methicillin-R** (N = 2) | **No data** (N = 2) | | | | |
| ***mecA* detection in tracheal aspirates (TA)** | *mecA* present (n, %) | 2 (33.3%) | 2 (100.0%) | 0 (0.0%) | **Sensitivity [CI]** | 1.00 [0.18-1.00] | **Negative predictive value** | 1.00 [0.51-1.00] |
| | *mecA* absent (n, %) | 4 (66.7%) | 0 (0.0%) | 2 (100.0%) | **Specificity [CI]** | 0.67 [0.30-0.94] | **Positive predictive value** | 0.5 [0.09-0.91] |
| | No TA samples available | 0 (0.0%) | 0 (0.0%) | 0 (0.0%) | | | | |
| | | | | | | | | |
| ***mecA* detection in nasal swabs (NS)** | *mecA* present (n, %) | 1 (16.7%) | 2 (100.0%) | 0 (0.0%) | **Sensitivity [CI]** | 1.00 [0.18-1.00] | **Negative predictive value** | 1.00 [0.51-1.00] |
| | *mecA* absent (n, %) | 4 (66.7%) | 0 (0.0%) | 2 (100.0%) | **Specificity [CI]** | 0.80 [0.38-0.99] | **Positive predictive value** | 0.67 [0.12-0.98] |
| | No NS samples available | 1 (16.7%) | 0 (0.0%) | 0 (0.0%) | | | | |

**Supplementary Table 4. Relationship between *CTX-M* (detection and phenotypic susceptibility to ceftriaxone in cultured *Enterobacteriaceae* isolates from patients with 2°BP. (N = 12 cultured isolates from 11 patients).** Detection of *CTX-M* genes from tracheal aspirate or nasal swab samples within -7/+7 days of 2°BP were compared with phenotypic ceftriaxone susceptibility of *Enterobacteriaceae* isolates cultured from tracheal aspirate. One patient had an isolate for which phenotypic susceptibility was not performed (No data). Isolates with susceptibilities reported as “susceptible dose dependent” (N =1 pathogen-antibiotic combination) or “intermediate” (N=2 pathogen-antibiotic combinations) were considered as resistant for the purposes of this analysis.

| | | **Clinical Susceptibility** | | | **Calculated test performance** | | | |
|---|---|---|---|---|---|---|---|---|
| | | **Ceftriaxone-S** (N = 5) | **Ceftriaxone-R** (N = 6) | **No data** (N = 1) | | | | |
| ***CTX-M* gene detection in tracheal aspirates (TA)** | Present (n, %) | 0 (0.0%) | 4 (66.7%) | 1 (100.0%) | **Sensitivity [CI]** | 0.67 [0.30-0.94] | **Negative predictive value** | 0.71 [0.36-0.95] |
| | Absent (n, %) | 5 (100.0%) | 2 (33.3%) | 0 (0.0%) | **Specificity [CI]** | 1.00 [0.57-1.00] | **Positive predictive value** | 1.00 [0.51-1.00] |
| | No TA samples available | 0 (0.0%) | 0 (0.0%) | 0 (0.0%) | | | | |
| | | | | | | | | |
| ***CTX-M* gene detection in nasal swabs (NS)** | Present (n, %) | 0 (0.0%) | 2 (33.3%) | 0 (0.%) | **Sensitivity [CI]** | 1.00 [0.051-1.00] | **Negative predictive value** | 1.00 [0.18-1.00] |
| | Absent (n, %) | 1 (20.0% | 0 (0.0%) | 1 (100.0%) | **Specificity [CI]** | 1.00 [0.18-1.00] | **Positive predictive value** | 1.00 [0.051-1.00] |
| | No NS samples available | 4 (80.0%) | 4 (66.7%) | 0 (0.0%) | | | | |

**Supplementary Table 5. Concordance of AMR gene detection in tracheal aspirate and nasal swab samples.** Detection of 14 pathogen-associated AMR genes in tracheal aspirate or paired nasal swab samples is tabulated, as is co-detection of the AMR gene in both sample types.

| | **Concordance of AMR gene detection in tracheal aspirate and nasal swab samples** | | | | |
|---|---|---|---|---|---|
| | Total patients with any gene detection | AMR gene detected at both sites | AMR gene detected only in TA | AMR gene detected only in NS | No available NS samples |
| ACT-MIR | 2 | 0 (0.0%) | 1 (50.0%) | 0 (0.0%) | 1 (50.0%) |
| AmpC | 7 | 1 (14.3%) | 2 (28.6%) | 0 (0.0%) | 4 (57.1%) |
| CTX-M | 3 | 1 (33.3%) | 0 (0.0%) | 0 (0.0%) | 2 (66.7%) |
| FONA-1 | 3 | 1 (33.3%) | 0 (0.0%) | 0 (0.0%) | 2 (66.7%) |
| MAL-CKO | 1 | 0 (0.0%) | 0 (0.0%) | 0 (0.0%) | 1 (100.0%) |
| OXA-1 | 2 | 0 (0.0%) | 0 (0.0%) | 0 (0.0%) | 2 (100.0%) |
| OXA-50 | 5 | 2 (40.0%) | 0 (0.0%) | 0 (0.0%) | 3 (60.0%) |
| OXY | 3 | 1 (33.3%) | 0 (0.0%) | 0 (0.0%) | 2 (66.7%) |
| RAHN-1 | 3 | 1 (33.3%) | 0 (0.0%) | 0 (0.0%) | 2 (66.7%) |
| SED-1 | 3 | 1 (33.3%) | 0 (0.0%) | 0 (0.0%) | 2 (66.7%) |
| Qnr-1 | 1 | 1 (100.0%) | 0 (0.0%) | 0 (0.0%) | 0 (0.0%) |
| MCR-1 | 1 | 1 (100.0%) | 0 (0.0%) | 0 (0.0%) | 0 (0.0%) |
| Bla-Z | 5 | 3 (60.0%) | 2 (40.0%) | 0 (0.0%) | 0 (0.0%) |
| MecA | 6 | 2 (33.3%) | 2 (33.3%) | 1 (16.7%) | 1 (16.7%) |

**Supplementary Table 6. Associations between AMR gene detection and in-hospital mortality.** Associations between detection of specific AMR genes in either nasal swab or tracheal aspirate metatranscriptomic data and in-hospital mortality were assessed by Fisher's exact test.

| **Associations between AMR gene detection and in-hospital patient mortality** | | | |
|---|---|---|---|
| *Patients with gram negative 2°BP pathogens* (N = 18) | | | |
| | Survivors (N =7) | Deceased (N =11) | |
| AMR gene | Prevalence | Prevalence | P value |
| ACT-MIR | 0 (0.0%) | 2 (18.2%) | 0.50 |
| AmpC | 2 (28.6%) | 5 (45.5%) | 0.64 |
| CTX-M | 2 (28.6%) | 1 (9.1%) | 0.53 |
| FONA-1 | 2 (28.6%) | 1 (9.1%) | 0.53 |
| MAL-CKO | 1 (14.3%) | (0.0%) | 0.39 |
| OXA-1 | 2 (28.6%) | (0.0%) | 0.14 |
| OXA-50 | 1 (14.3%) | 4 (36.4%) | 0.60 |
| OXY | 2 (28.6%) | 1 (9.1%) | 0.53 |
| RAHN-1 | 2 (28.6%) | 1 (9.1%) | 0.53 |
| SED-1 | 2 (28.6%) | 1 (9.1%) | 0.53 |
| Qnr-1 | 0 (0.0%) | 1 (9.1%) | 1.00 |
| MCR-1 | 0 (0.0%) | 1 (9.1%) | 1.00 |
| *Patients with gram positive 2°BP pathogens (*N = 11) | | | |
| | Survivors (N = 6) | Deceased (N = 5) | |
| AMR gene | Prevalence | Prevalence | P value |
| Bla-Z | 2 (33.3%) | 3 (60.0%) | 0.57 |
| MecA | 3 (50.0%) | 3 (60.0%) | 1.00 |

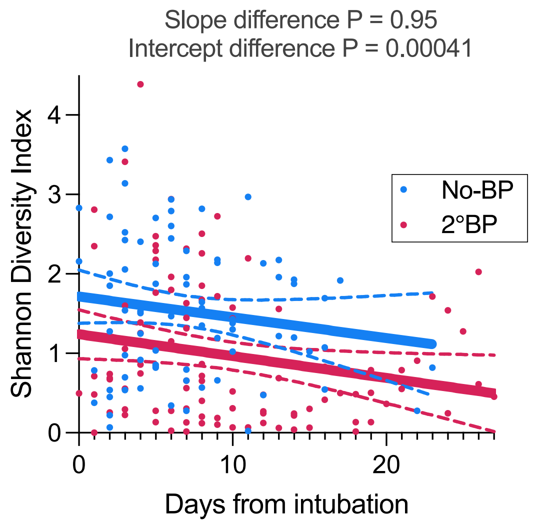


**Supplementary Figure 1. Changes in lower respiratory microbiome alpha diversity over time.** Linear regression model of all samples from 2°BP patients (red dots; N = 27 patients with a total of 99 samples) and No-BP patients (blue dots; N = 29 patients with a total of 79 samples). Slope and Y-intercept are shown with 95% confidence intervals.

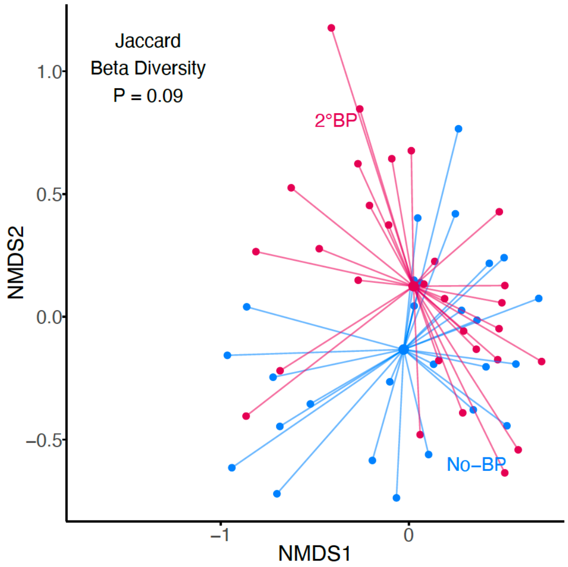


**Supplementary Figure 2. Beta diversity comparing tracheal aspirate samples from 2°BP versus No-BP patients, by Jaccard index.** Non-metric multidimensional scaling (NMDS) plot demonstrating compositional differences in the lung microbiome of 2°BP (red dots, N = 27) versus No-BP (blue dots, N = 29) patients based on Jaccard dissimilarity index and the PERMANOVA test with 1000 permutations.

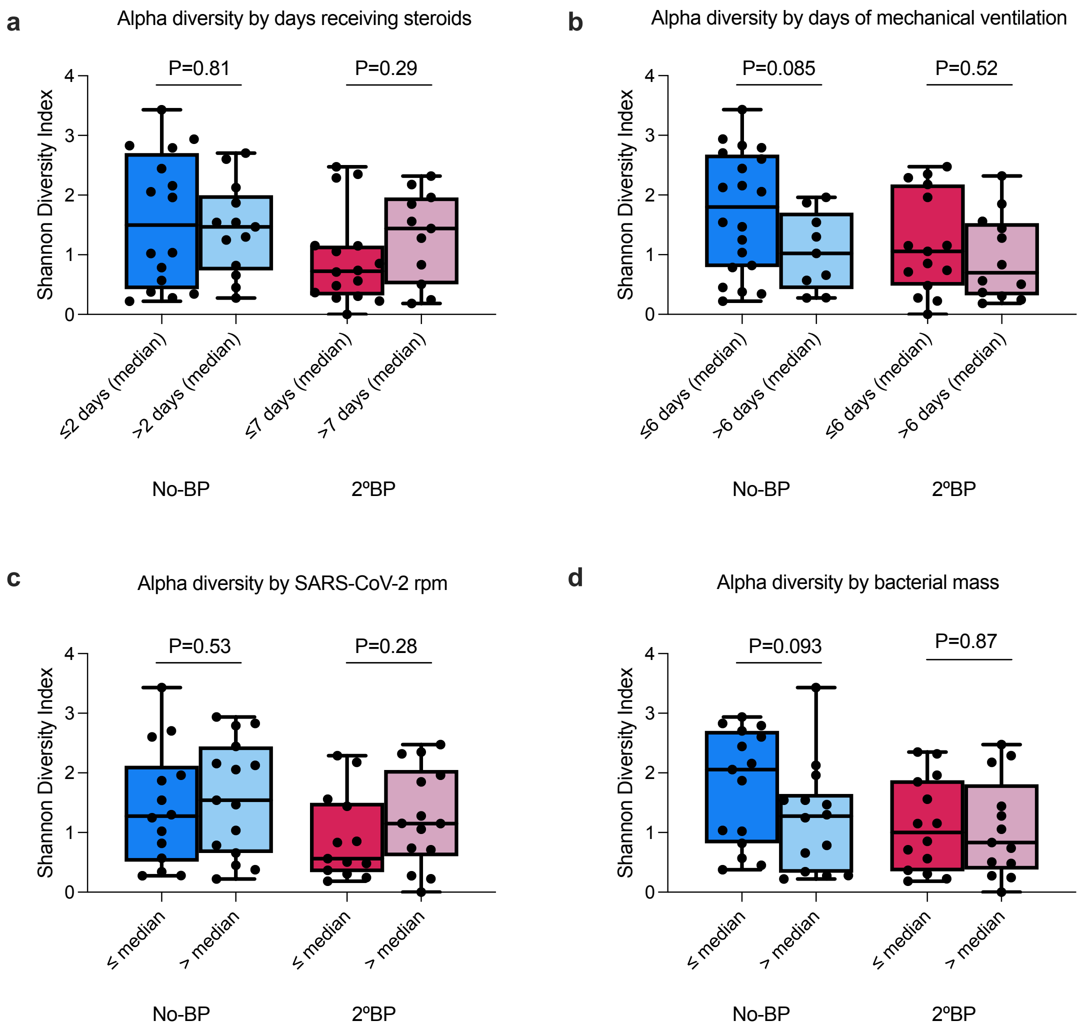


**Supplementary Figure 3. Alpha diversity based on days of steroid receipt, days of mechanical ventilation, SARS-CoV-2 viral load and bacterial mass.** Analysis performed comparing alpha diversity between patients with No-BP (left columns, blue, N = 29) and 2°BP (right columns, red, N = 27) as compared by patients who were **a** above or below median days of steroid receipt (2 days in No-BP, 7 days in 2°BP), **b** above or below median number of mechanical ventilation days (6 days in both groups), **c** above or below the median SARS-CoV-2 viral load (93.2 rpM in No-BP, 15.9 rpm in 2°BP), **d** above or below the median bacterial mass (5.1 pg in No-BP, 90.9 pg in 2°BP). Boxes show median and 25$^{th}$-75$^{th}$ percentiles, with whiskers from min to max. P values calculated by two-sided Wilcoxon tests.

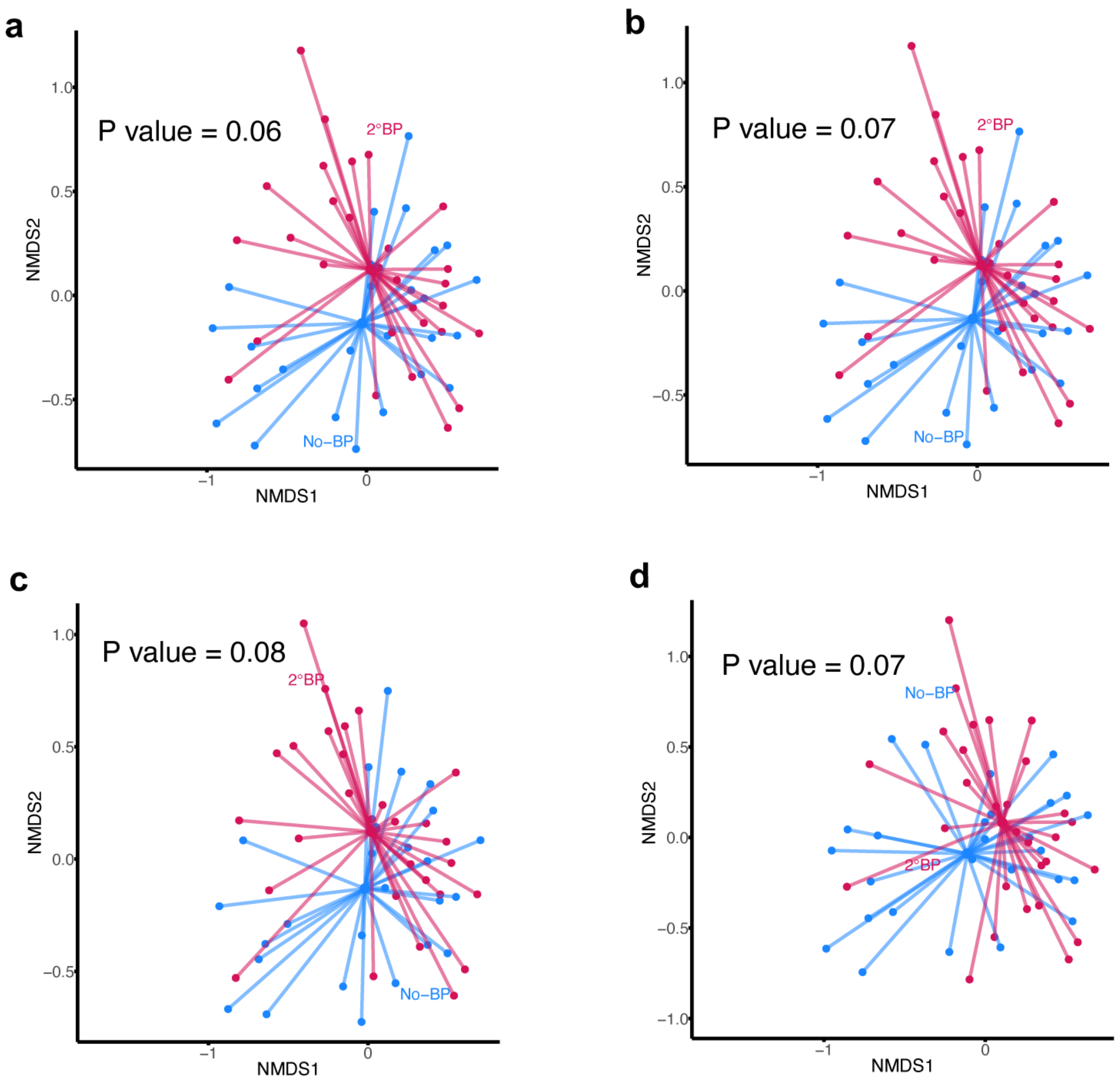


**Supplementary Figure 4. Beta diversity comparing tracheal aspirate samples from 2°BP versus No-BP patients, adjusted for days of steroid receipt, days of mechanical ventilation, SARS-CoV-2 viral load or bacterial mass**. Non-metric multidimensional scaling (NMDS) plots demonstrating beta diversity based on Bray-Curtis dissimilarity index, adjusted for **a** days of steroid receipt, **b** days of mechanical ventilation, **c** SARS-CoV-2 reads per million, **d** bacterial mass**.** P values based on the PERMANOVA test with 1000 permutations. Red dots are patients with 2°BP (N = 27); blue dots are No-BP (N = 29)

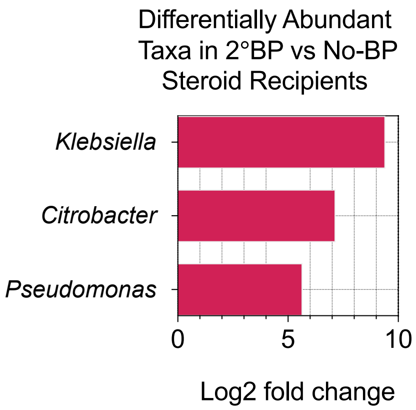


**Supplementary Figure 5. Differential bacterial abundance based on 2°BP status, limited to patients who received steroids.** Red bars indicate increased relative abundance of genera in 2°BP patients (N = 25), blue bars indicate increased relative abundance in No-BP patients (N = 19). Bacterial taxa are shown if Padj < 0.001, and positive log fold change indicates enrichment for microbes in patients with 2°BP as compared to No-BP

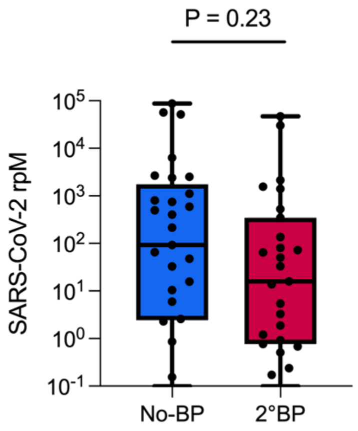


**Supplementary Figure 6. SARS-CoV-2 viral load based on 2°BP status.** Viral load measured in reads per million (rpM) in 2°BP (red, N = 27) or No-BP (blue, N = 29) patients. Boxes show median and 25$^{th}$-75$^{th}$ percentiles, with whiskers from min to max. P value calculated by two-sided Wilcoxon test.

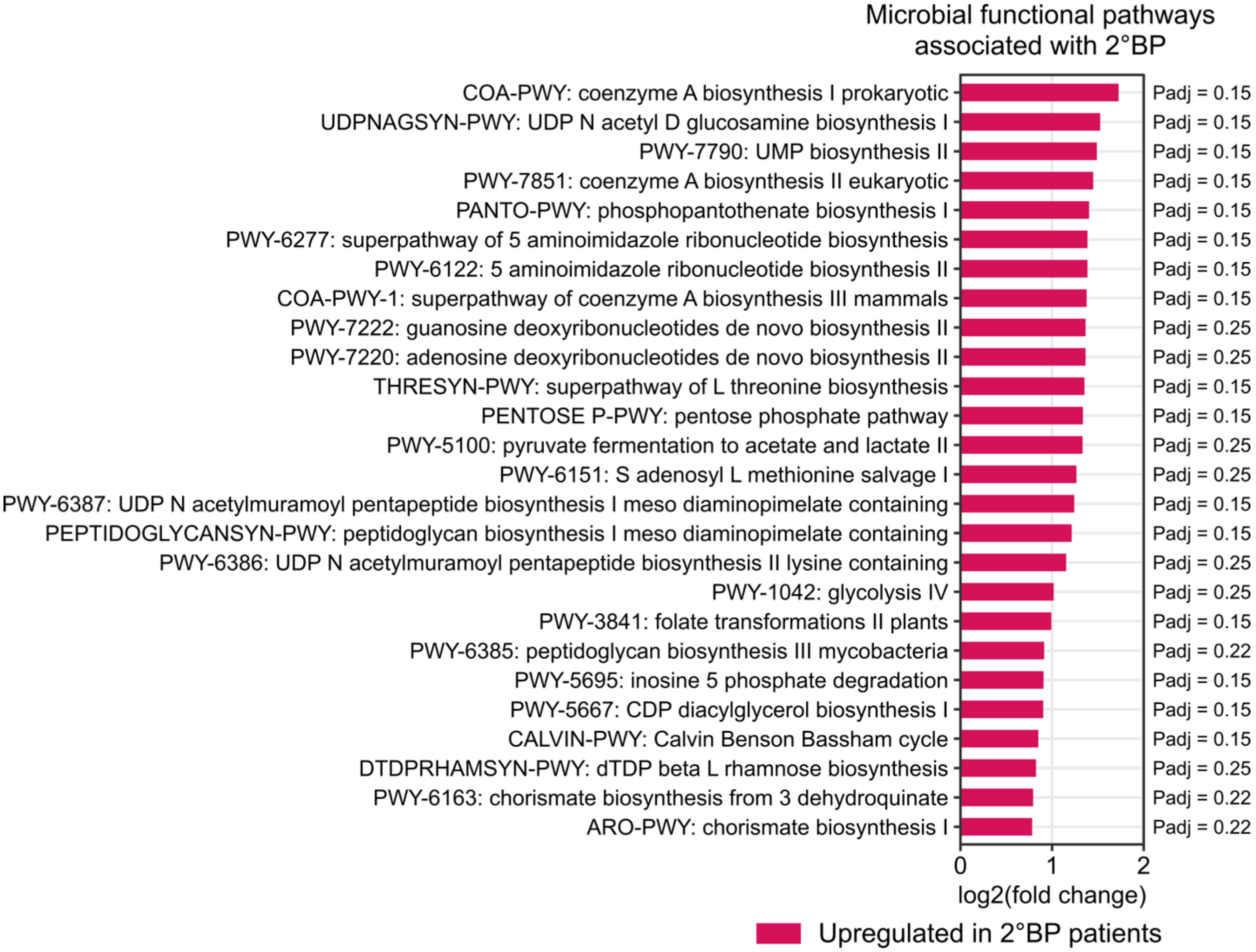


**Supplementary Figure 7. Functional analysis of bacterial metabolic pathways based on 2°BP status**. Metabolic pathways differentially abundant in 2°BP patients (N = 27) versus No-BP patients (N = 29) are highlighted. All pathways detected at false discovery rate < 0.25 are included in plot.

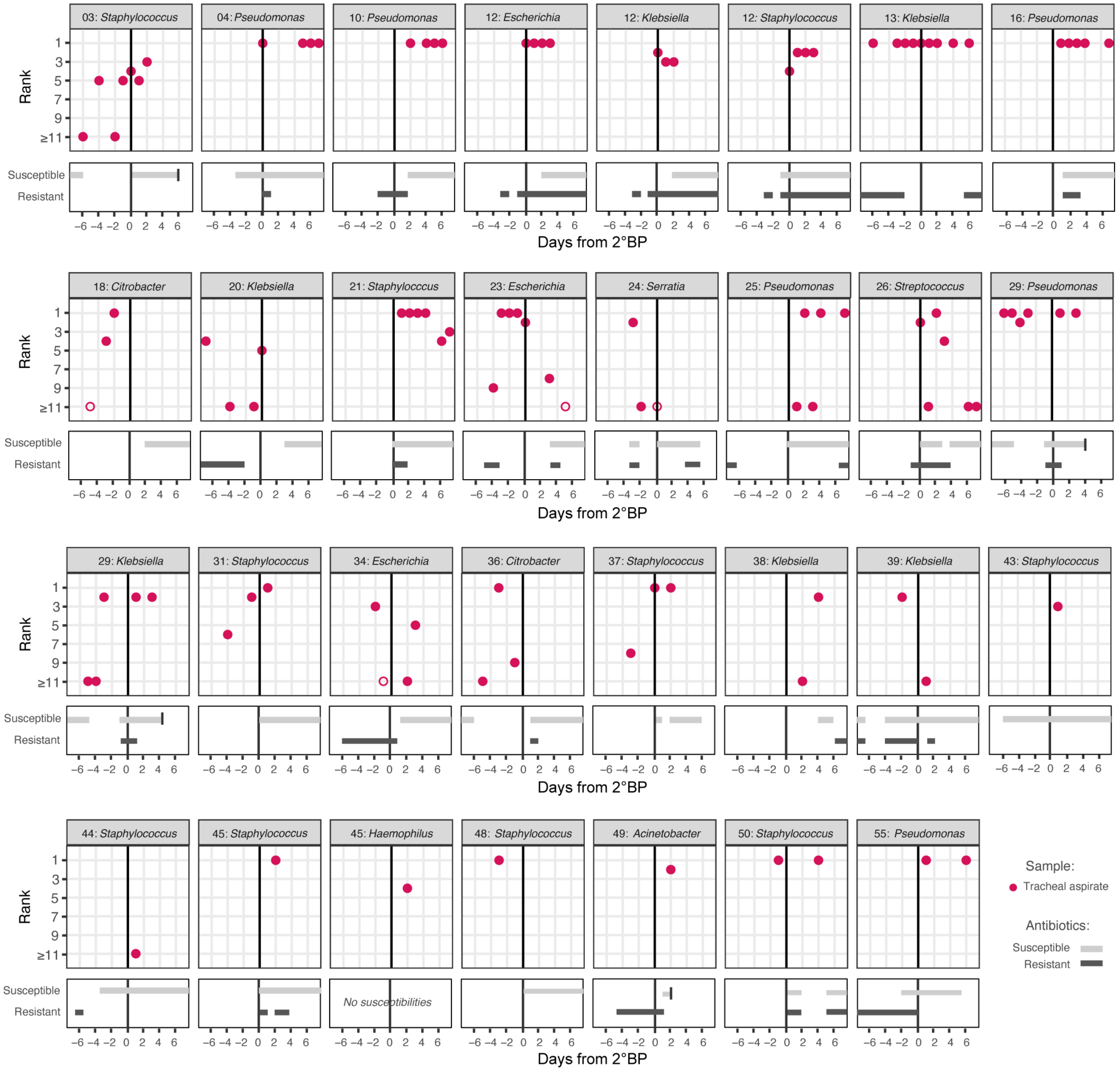


**Supplementary Figure 8. Pathogen rank plots of tracheal aspirate samples**. Genus level 2°BP pathogen rank based on bacterial reads per million (rpM) in the lung microbiome. Days relative to 2°BP clinical diagnosis on the X axis. Days during which patient received antibiotics to which 2°BP pathogen was phenotypically susceptible (light grey bar) or resistant (dark grey bar) shown below. Patient death during this period is shown using a vertical black bar. Patient ID and pathogen genus are listed above each plot. N = 27 patients. For patients with multiple cultured pathogens (12, 29 and 45), each plot represents a cultured pathogen. Open circles indicate that the pathogen was not detected in a sample.

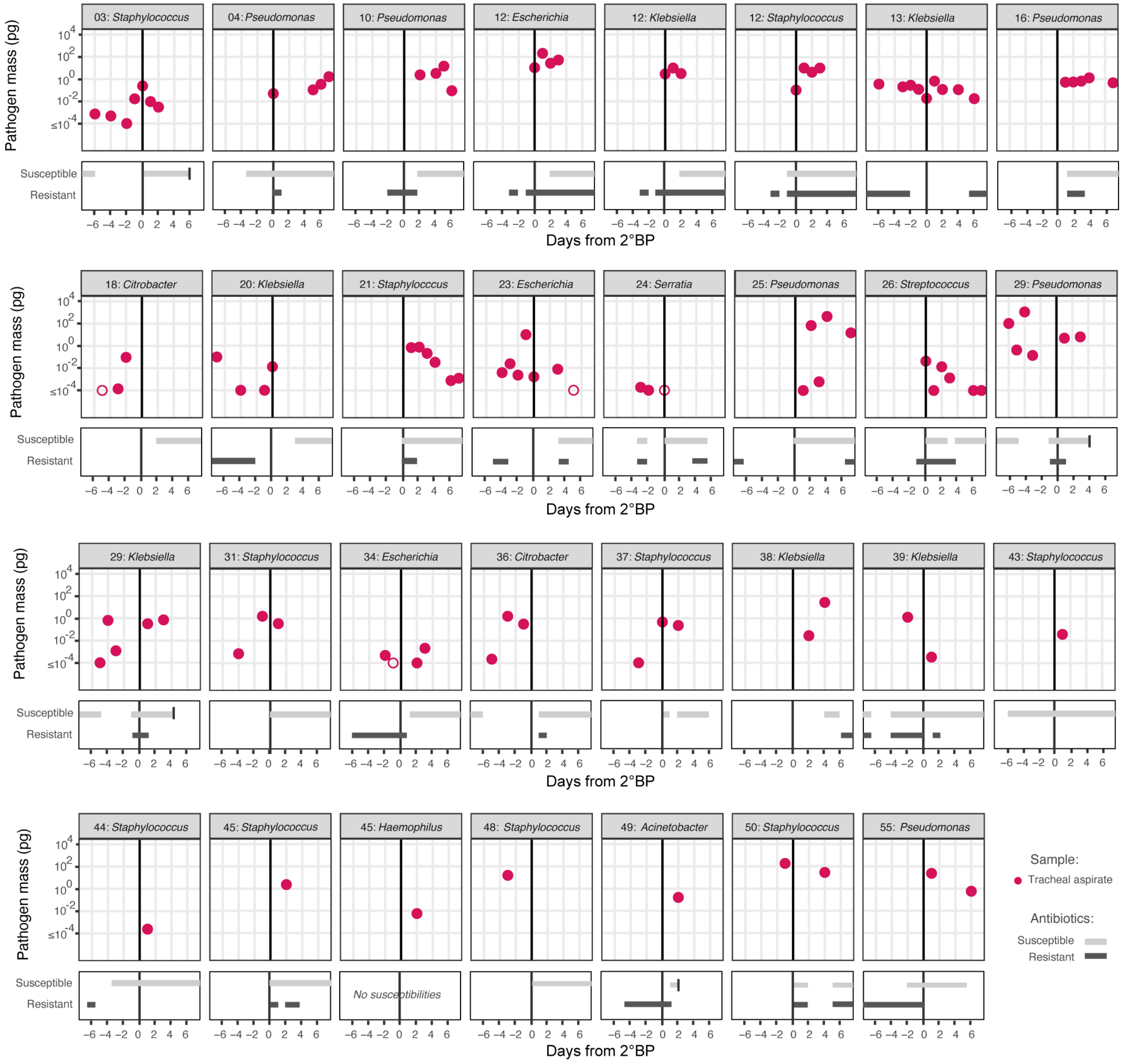


**Supplementary Figure 9. Pathogen mass plots of tracheal aspirate samples**. Pathogen mass in picograms (pg) by pathogen reads as compared to spiked-in mass controls. Days relative to 2°BP clinical diagnosis on the X axis. Days during which patient received antibiotics to which 2°BP pathogen was phenotypically susceptible (light grey bar) or resistant (dark grey bar) shown below. Patient death during this period is shown using a vertical black bar. Patient ID and pathogen genus are listed above each plot. N = 27 patients. For patients with multiple cultured pathogens (12, 29 and 45), each plot represents a cultured pathogen. Open circles indicate that the pathogen was not detected in a sample.

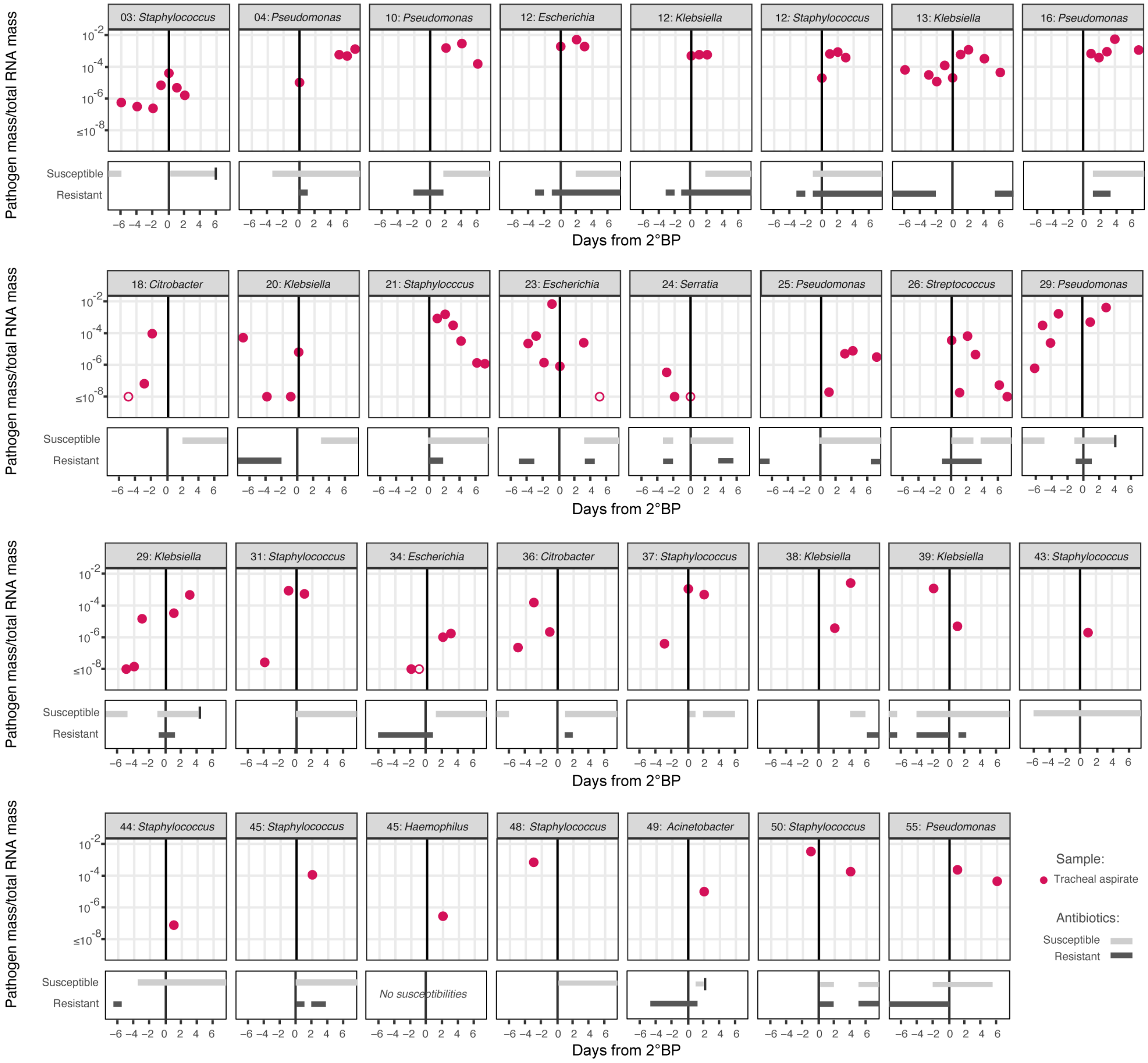


**Supplementary Figure 10. Pathogen normalized mass plots of tracheal aspirate samples.** Pathogen mass is shown normalized to the total RNA mass of sample. Days relative to 2°BP clinical diagnosis on the X axis. Days during which patient received antibiotics to which 2°BP pathogen was phenotypically susceptible (light grey bar) or resistant (dark grey bar) shown below. Patient death during this period is shown using a vertical black bar. Patient ID and pathogen genus are listed above each plot. N = 27 patients. For patients with multiple cultured pathogens (12, 29 and 45), each plot represents a cultured pathogen. Open circles indicate that the pathogen was not detected in a sample.

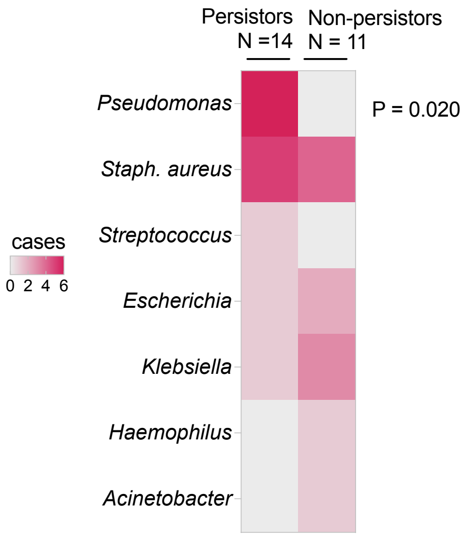


**Supplementary Figure 11. Comparative persistence of 2°BP pathogens in tracheal aspirate samples**. Heatmap demonstrating the number of cases in which the culture-confirmed 2°BP pathogen remained top-ranked (based on abundance) in the lung microbiome for at least one day following clinical diagnosis of 2°BP (persistors) versus those which did not exhibit impaired clearance (non-persistors). P value based on Fisher's exact test comparing *Pseudomonas* to all other pathogens combined. N = 25 patients with available samples after clinical 2°BP diagnosis.

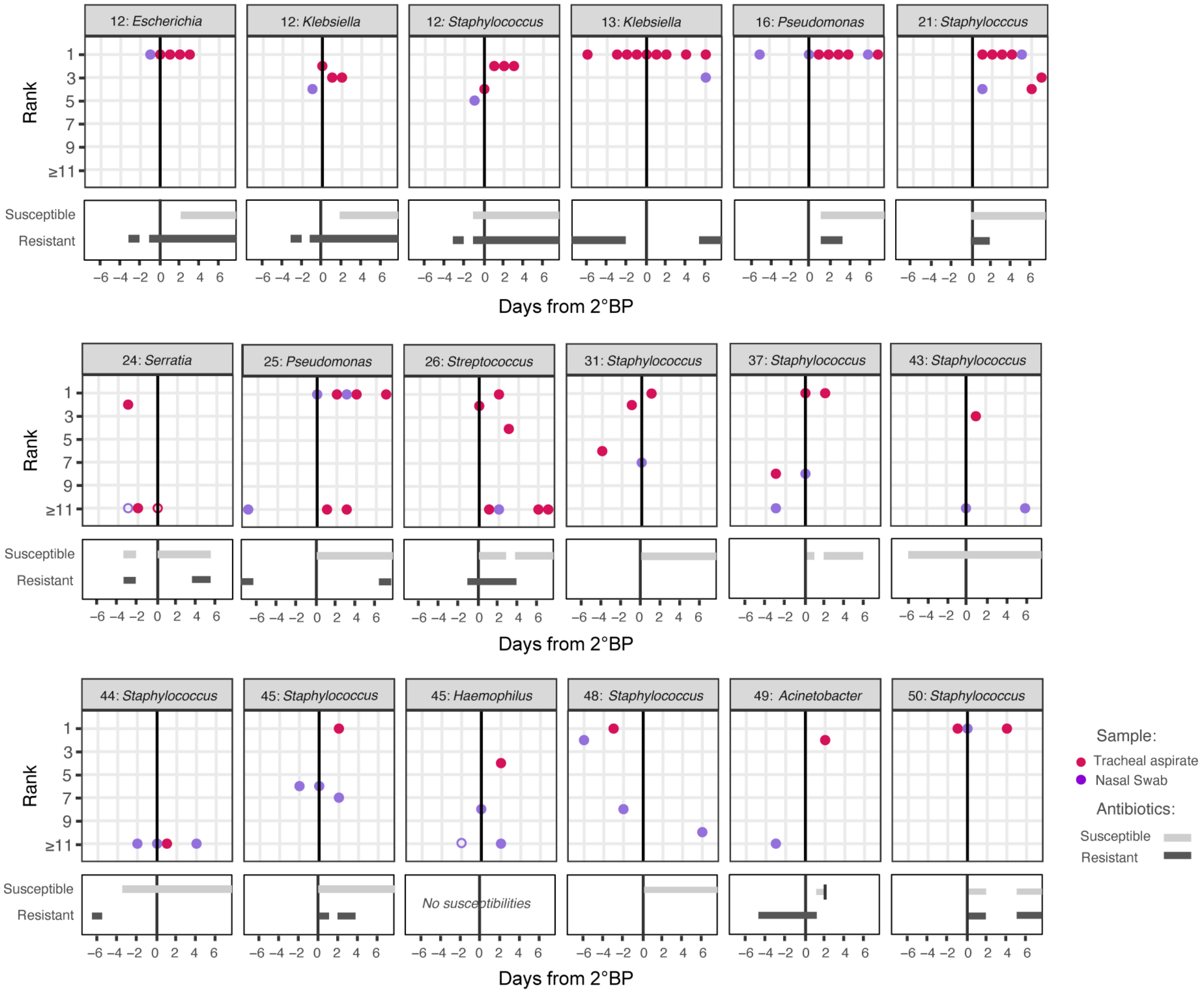


**Supplementary Figure 12. Pathogen rank plots of nasal swab and tracheal aspirate samples**. Genus level 2°BP pathogen rank based on bacterial reads per million (rpM) in the lung microbiome (red) or nares microbiome (purple). Only patients with nasal swab data are shown. Days relative to 2°BP clinical diagnosis on the X axis. Days during which patient received antibiotics to which 2°BP pathogen was phenotypically susceptible (light grey bar) or resistant (dark grey bar) shown below. Patient death during this period is shown using a vertical black bar. Patient ID and pathogen genus are listed above each plot. N = 15 patients. For patients with multiple cultured pathogens (12 and 45), each plot represents a cultured pathogen. Open circles indicate that the pathogen was not detected in a sample.

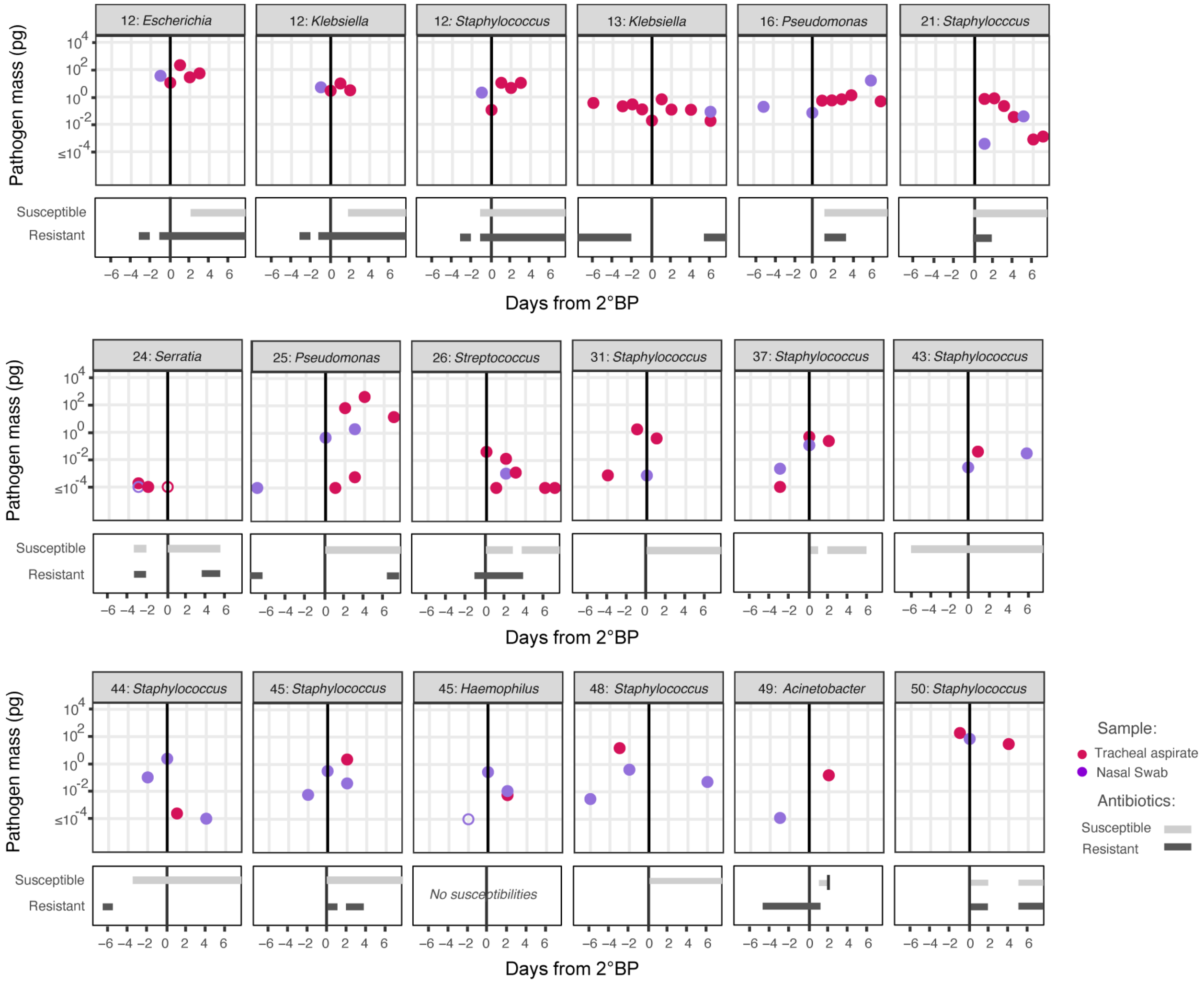


**Supplementary Figure 13. Pathogen mass plots of nasal swab and tracheal aspirate samples.** Pathogen mass as calculated in picograms (pg) by pathogen reads as compared to spiked-in mass controls and shown in the lung microbiome (red) or nares microbiome (purple). Only patients with nasal swab data are shown. Days relative to 2°BP clinical diagnosis on the X axis. Days during which patient received antibiotics to which 2°BP pathogen was phenotypically susceptible (light grey bar) or resistant (dark grey bar) shown below. Patient death during this period is shown using a vertical black bar. Patient ID and pathogen genus are listed above each plot. N = 15 patients. For patients with multiple cultured pathogens (12 and 45), each plot represents a cultured pathogen. Open circles indicate that the pathogen was not detected in a sample.

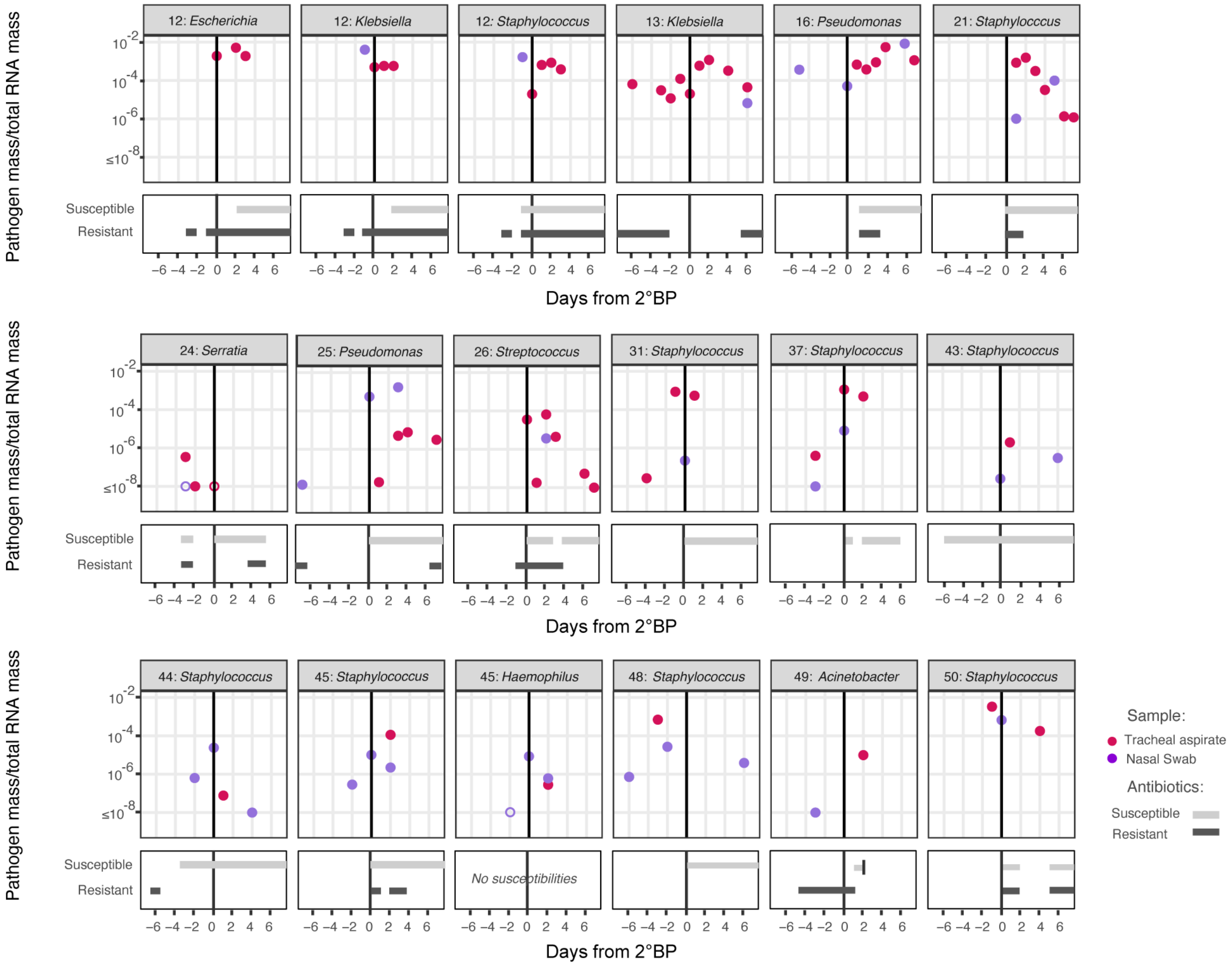


**Supplementary Figure 14. Pathogen normalized mass plots of nasal swab and tracheal aspirate samples.** Pathogen mass in the nares (purple) or tracheal aspirate (red) is shown as normalized to the total RNA mass of sample. Only patients with nasal swab data are shown. Days relative to 2°BP clinical diagnosis on the X axis. Days during which patient received antibiotics to which 2°BP pathogen was phenotypically susceptible (light grey bar) or resistant (dark grey bar) shown below. Patient death during this period is shown using a vertical black bar. Patient ID and pathogen genus are listed above each plot. N = 15 patients. For patients with multiple cultured pathogens (12 and 45), each plot represents a cultured pathogen. Open circles indicate that the pathogen was not detected in a sample.

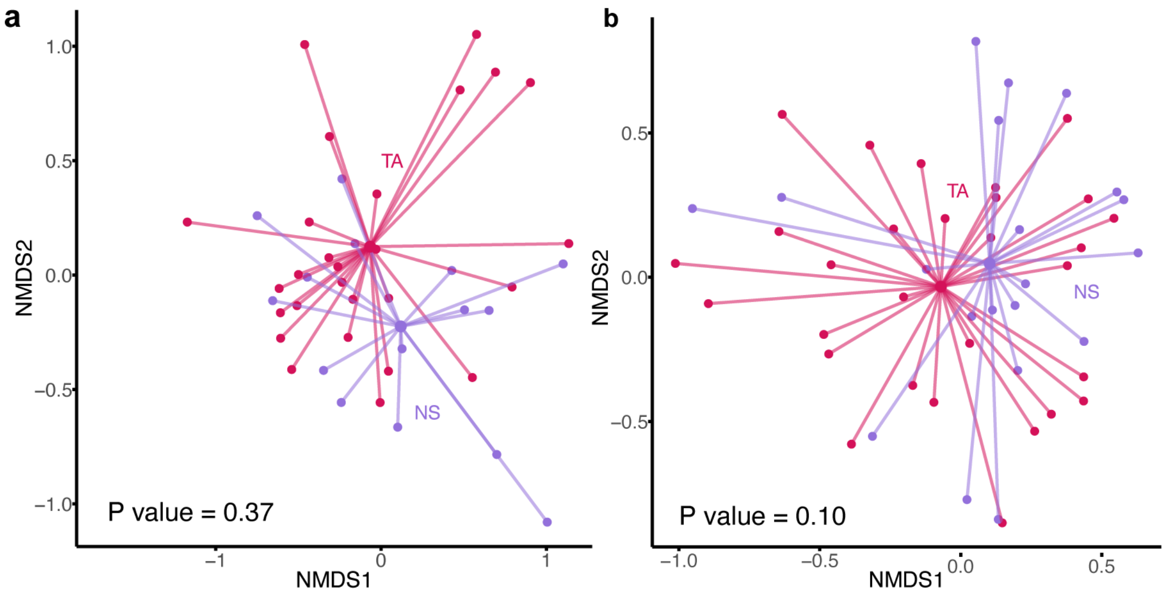


**Supplementary Figure 15. Beta diversity comparing nasal swab to tracheal aspirate samples.** Non-metric multidimensional scaling (NMDS) plots demonstrating upper versus lower airway beta diversity based on Bray-Curtis dissimilarity index between nasal swabs (NS, purple) and tracheal aspirate (TA, red), accounting for intra-participant clustering. **a** 2°BP patients (N = 15) and **b** No-BP patients (N = 20). P values based on the PERMANOVA test with 1000 permutations.

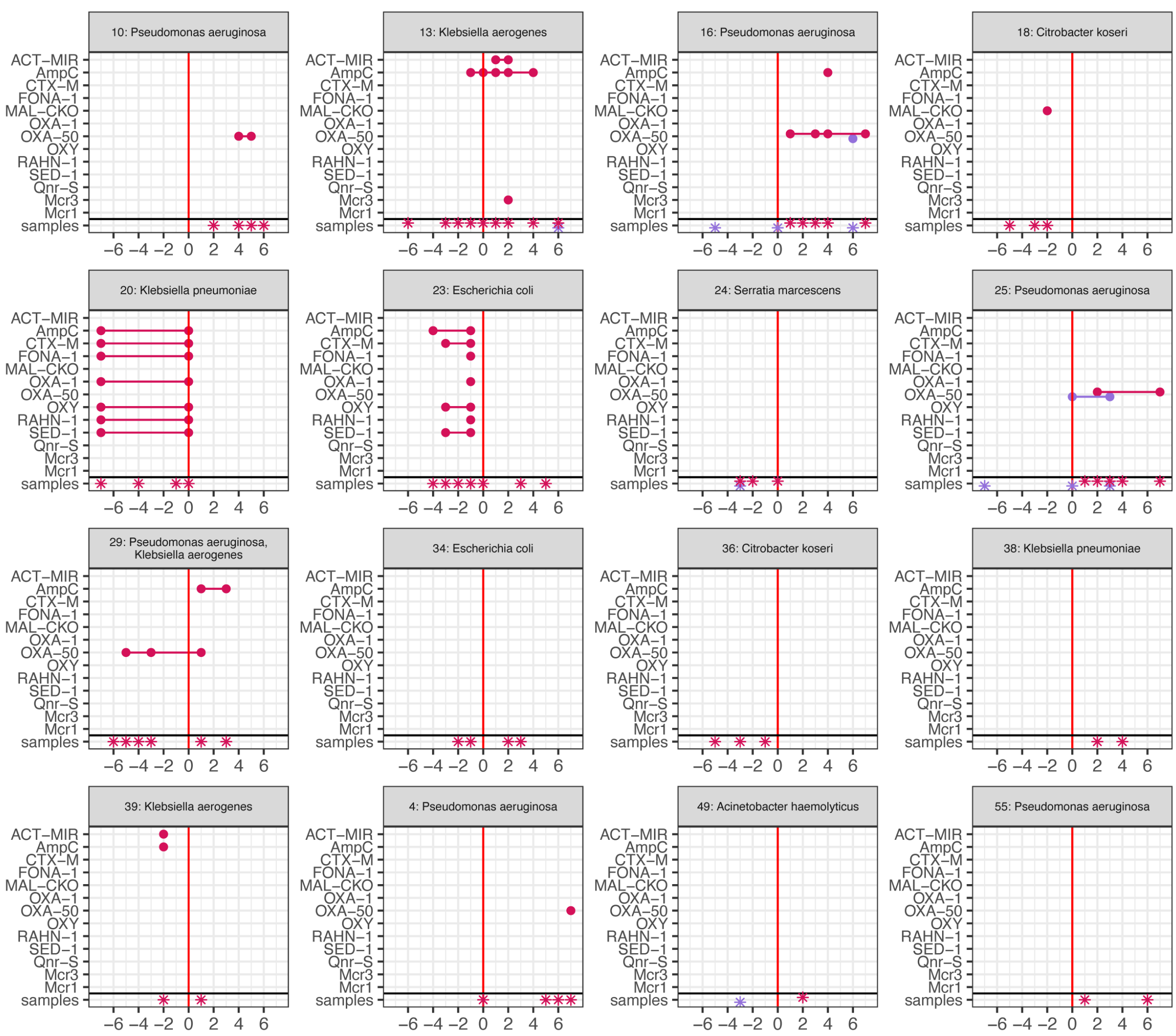
a
Specimen sample
TA
Nasal Swab
10: Pseudomonas aeruginosa
13: Klebsiella aerogenes
16: Pseudomonas aeruginosa
18: Citrobacter koseri
20: Klebsiella pneumoniae
23: Escherichia coli
24: Serratia marcescens
25: Pseudomonas aeruginosa
29: Pseudomonas aeruginosa, Klebsiella aerogenes
34: Escherichia coli
36: Citrobacter koseri
38: Klebsiella pneumoniae
39: Klebsiella aerogenes
4: Pseudomonas aeruginosa
49: Acinetobacter haemolyticus
55: Pseudomonas aeruginosa
ACT−MIR
AmpC
CTX−M
FONA−1
MAL−CKO
OXA−1
OXA−50
OXY
RAHN−1
SED−1
Qnr−S
Mcr3
Mcr1
samples
−6 −4 −2 0 2 4 6

**b**

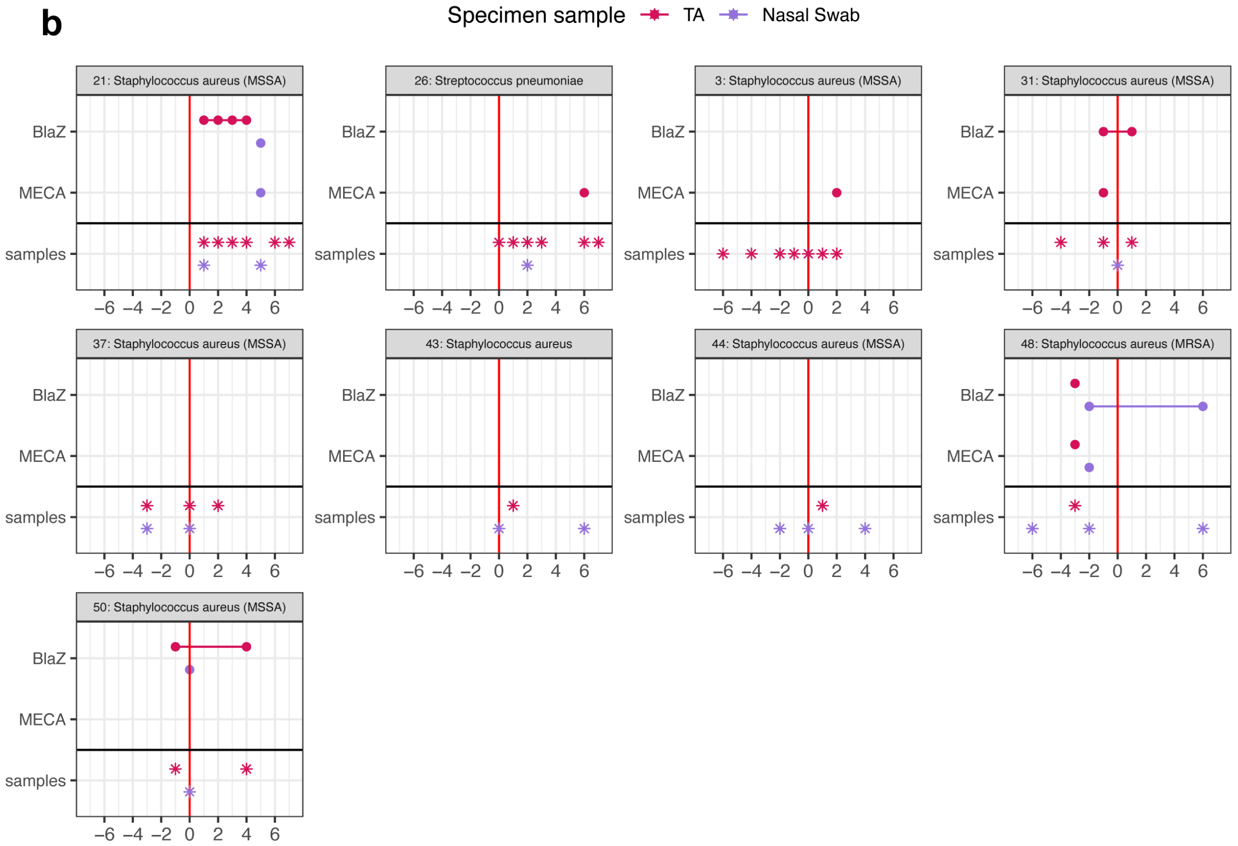


**c**

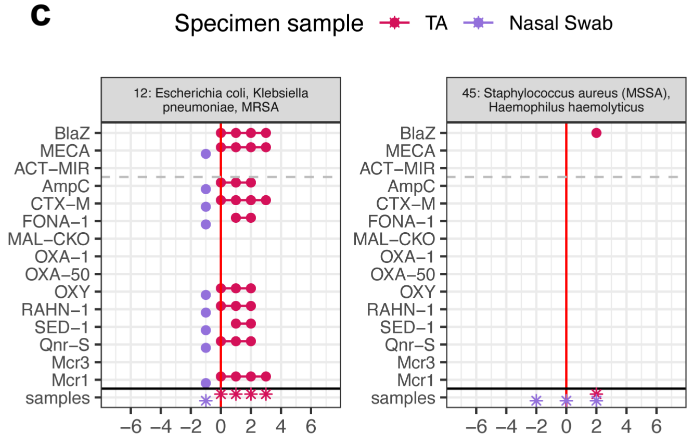


**Supplementary Figure 16. Per-patient identification of pathogen-associated antimicrobial resistance genes.** Longitudinal dynamics of 2°BP pathogen-associated antimicrobial resistance (AMR) genes of clinical and public health significance detected in tracheal aspirate (red) or nasal swab (purple) metatranscriptomic data. AMR genes are plotted based on association with **a** gram negative 2°BP pathogens (N = 16 patients), **b** gram positive 2°BP pathogens (N = 9 patients), or **c** mixed gram-positive and gram-negative infections (N = 2 patients). Samples available for analysis are noted (*), immediately above the x-axis.

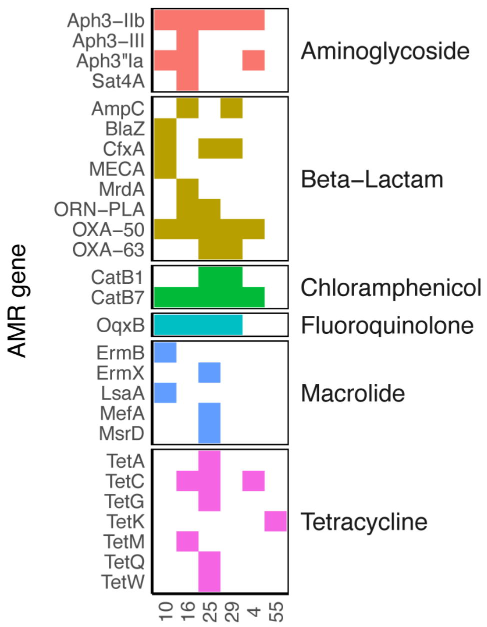


**Supplementary Figure 17. Antimicrobial resistance (AMR) genes detected in patients with *Pseudomonas aeruginosa* 2°BP*.*** AMR genes detected in tracheal aspirate (TA) within 7 days of 2°BP clinical diagnosis grouped and colored by class. N = 6 patients.
