# Supplementary material for: Microbial dynamics and pulmonary immune responses in COVID-19 secondary bacterial pneumonia
Source: Nat Commun. 2024 Oct 29;15:9339. doi: 10.1038/s41467-024-53566-x (PMC11522429; doi:10.1038/s41467-024-53566-x)
Supplement: Supplementary file 3 — Description of Additional Supplementary Files [file 41467_2024_53566_MOESM3_ESM.pdf]

Description of Additional Supplementary Files

Supplementary Data 1. Antimicrobial susceptibility patterns of cultured microbes.

Supplementary Data 2. Functional analysis of metabolic pathways identified in the lower airway microbiome, compared between 2°BP patients and No-BP controls.

Supplementary Data 3. Differentially expressed genes between 2°BP patients and No-BP controls in tracheal aspirates. Legend: LogFC = log2 fold change; pval = P value; padj = adjusted P value.

Supplementary Data 4. Gene set enrichment analysis based on differentially expressed genes between 2°BP patients and No-BP controls from tracheal aspirate metatranscriptomics. Legend: pval = P value; padj = adjusted P value; NES = normalized enrichment score.

Supplementary Data 5. Differentially expressed genes between 2°BP patients and No-BP controls from tracheal aspirate metatranscriptomics, restricted to a sub-analysis of patients who received treatment with corticosteroids. Legend: LogFC = log2 fold change; pval = P value; padj = adjusted P value.

Supplementary Data 6. Gene set enrichment analysis of genes differentially expressed between 2°BP patients and No-BP controls, restricted to a sub-analysis of only patients who received treatment with corticosteroids. Legend: pval = P value; padj = adjusted P value; NES = normalized enrichment score.

Supplementary Data 7. Gene set enrichment analysis of genes differentially expressed based on days of steroid receipt in patients with 2°BP, limited to patients who received any steroids. Legend: pval = P value; padj = adjusted P value; NES = normalized enrichment score.

Supplementary Data 8. Gene set enrichment analysis of genes differentially expressed based on days of steroid receipt in patients with No-BP, limited to patients who received any steroids. Legend: pval = P value; padj = adjusted P value; NES = normalized enrichment score.

Supplementary Data 9. Genes differentially expressed in 2°BP patients based on bacterial RNA mass. Legend: LogFC = log2 fold change; pval = P value; padj = adjusted P value.

Supplementary Data 10. Genes differentially expressed in No-BP patients based on bacterial RNA mass. Legend: LogFC = log2 fold change; pval = P value; padj = adjusted P value.

Supplementary Data 11. Gene set enrichment analysis of genes differentially expressed in 2°BP patients based on bacterial RNA mass. Legend: pval = P value; padj = adjusted P value; NES = normalized enrichment score.

Supplementary Data 12. Gene set enrichment analysis of genes differentially expressed in No-BP patients based on bacterial RNA mass. Legend: pval = P value; padj = adjusted P value; NES = normalized enrichment score.

Supplementary Data 13. Gene set enrichment analysis of genes differentially expressed in 2°BP patients based on bacterial RNA mass, limited to patients who received steroids. Legend: pval = P value; padj = adjusted P value; NES = normalized enrichment score.

Supplementary Data 14. Gene set enrichment analysis of genes differentially expressed in No-BP patients based on bacterial RNA mass, limited to patients who received steroids. Legend: pval = P value; padj = adjusted P value; NES = normalized enrichment score.
